# Effects of Nasoalveolar Molding on Maxillary Arch Dimensions and Morphology in Unilateral and Bilateral Cleft Lip and Palate: A Systematic Review and Meta-Analysis

**DOI:** 10.21142/2523-2754-1404-2026-321

**Published:** 2026-10-01

**Authors:** Arezoo Jahanbin, Alireza Sarraf Shirazi, Haniye Atashkar, Farzaneh Lal Alizadeh

**Affiliations:** 1 Department of Orthodontics, School of Dentistry, Mashhad University of Medical Sciences. Mashhad, Iran. dr_arezoo_jahanbin@yahoo.com fla7989@gmail.com Mashhad University of Medical Sciences Department of Orthodontics School of Dentistry Mashhad University of Medical Sciences Mashhad Iran dr_arezoo_jahanbin@yahoo.com fla7989@gmail.com; 2 Department of Pediatric Dentistry, School of Dentistry, Mashhad University of Medical Sciences. Mashhad, Iran. sarrafa@mums.ac.ir Mashhad University of Medical Sciences Department of Pediatric Dentistry School of Dentistry Mashhad University of Medical Sciences Mashhad Iran sarrafa@mums.ac.ir; 3 Private Practice in Dentistry. hanieatashkar@gmail.com Private Practice in Dentistry hanieatashkar@gmail.com

**Keywords:** nasoalveolar molding, presurgical orthopedics, maxillary arch morphology, unilateral cleft lip and palate, bilateral cleft lip and palate, moldeo nasoalveolar, ortopedia prequirúrgica, morfología del arco maxilar, labio y paladar hendido unilateral, labio y paladar hendido bilateral

## Abstract

**Objective:**

The aim of this study was to determine whether nasoalveolar molding (NAM) produces temporary improvement or stable changes in maxillary arch growth and orientation.

**Materials and Methods:**

A systematic search of ISI (Web of Science), PubMed, Scopus, EMBASE, and Cochrane CENTRAL databases was conducted for studies indexed up to July 2025 following PRISMA guidelines. Six eligible studies were included, five on unilateral cleft lip and palate (UCLP) and one on bilateral cleft lip and palate (BCLP). Data on Anterior Arch Cleft Width (AACW), Posterior Arch Width (PAW), and Intercanine Width (ICW) were extracted and pooled for meta analysis. Standardized Mean Difference (SMD) was calculated, and a random effects model was applied when heterogeneity was present. Statistical significance was set at p < 0.05.

**Results:**

NAM produced notable short term changes in maxillary arch morphology before lip surgery. Most studies reported a significant reduction in anterior cleft width (AACW) in the NAM group compared with controls. Trends toward decreased intercanine width (ICW) and increased posterior arch width (PAW) were also observed, although these changes were not consistently statistically significant. No meaningful differences were found for middle arch width (MAW) or alveolar arch length (AAL). One long term follow up study suggested that some structural improvements may persist for several years, although natural growth, subsequent surgeries, and individual variability may influence outcomes. Meta analysis confirmed a significant short term reduction in AACW (SMD = 4.105, 95% CI = 1.961-6.249, p = 0.000), while no significant effects were found for other parameters. The reduction in AACW was less pronounced in BCLP than in UCLP patients.

**Conclusion:**

This study showed that NAM significantly reduces anterior cleft width before lip surgery, while its effects on other arch dimensions remain inconsistent. Evidence for bilateral cleft lip and palate and long term outcomes is limited, highlighting the need for further high quality research.

## RESUMEN

**Objetivo:**

Determinar si el moldeo nasoalveolar (MNA) produce una mejoría temporal o cambios estables en el crecimiento y la orientación del arco maxilar.

**Materiales y métodos:**

Se realizó una búsqueda sistemática en las bases de datos ISI (Web of Science), PubMed, Scopus, EMBASE y Cochrane CENTRAL para identificar estudios indexados hasta julio de 2025, siguiendo las directrices PRISMA. Se incluyeron seis estudios elegibles: cinco sobre labio y paladar hendido unilateral (LPHU), y uno sobre labio y paladar hendido bilateral (LPHB). Se extrajeron y agruparon los datos sobre el ancho del arco anterior (AAA), el ancho del arco posterior (AAP) y el ancho intercanino (AIC) para su metaanálisis. Se calculó la diferencia de medias estandarizada (DME) y se aplicó un modelo de efectos aleatorios cuando se detectó heterogeneidad. La significación estadística se estableció en p < 0,05.

**Resultados:**

El MNA produjo cambios notables a corto plazo en la morfología del arco maxilar antes de la cirugía labial. La mayoría de los estudios reportaron una reducción significativa del ancho de la hendidura anterior (AACW) en el grupo NAM en comparación con el grupo de control. También se observaron tendencias a una disminución del ancho intercanino (ICW) y a un aumento del ancho del arco posterior (PAW), aunque estos cambios no fueron estadísticamente significativos de manera consistente. No se encontraron diferencias significativas en el ancho del arco medio (MAW) ni en la longitud del arco alveolar (AAL). Un estudio de seguimiento a largo plazo sugirió que algunas mejoras estructurales pueden persistir durante varios años, si bien el crecimiento natural, las cirugías posteriores y la variabilidad individual pueden influir en los resultados. El metaanálisis confirmó una reducción significativa a corto plazo en el AACW (DME = 4,105, IC del 95 % = 1,961-6,249, p = 0,000), mientras que no se encontraron efectos significativos en otros parámetros. La reducción en el AACW fue menos pronunciada en pacientes con labio leporino bilateral (BCLP) que en pacientes con labio leporino unilateral (UCLP).

**Conclusión:**

Este estudio demostró que el NAM reduce significativamente el ancho de la hendidura anterior antes de la cirugía labial, mientras que sus efectos en otras dimensiones del arco siguen siendo inconsistentes. La evidencia sobre el labio y el paladar hendidos bilaterales y sus resultados a largo plazo es limitada, lo que subraya la necesidad de realizar más investigaciones de alta calidad.

## INTRODUCTION

Cleft lip and palate is one of the most frequent congenital craniofacial malformations, with a global incidence ranging between 1 in 700 live births. ^1^ This anomaly disrupts essential functions such as feeding, speech, breathing, and facial growth, and requires multidisciplinary care from birth onward. ^2^ Management of Cleft lip and palate typically involves staged surgical corrections; however, the severity of the deformity and the position of maxillary segments before surgery strongly influence surgical outcomes and long-term facial development. ^3^^,^^4^

Presurgical infant orthopedics has been introduced to improve the alignment of maxillary segments prior to primary lip repair. ^5^ Among the various approaches, nasoalveolar molding (NAM) has gained considerable attention since it not only narrows the alveolar cleft gap but also molds nasal cartilage, thereby improving symmetry and facilitating subsequent surgical procedures. ^6^^,^^7^

While NAM has become widely adopted, controversies remain regarding its true effectiveness. Some studies report significant improvements in alveolar arch morphology, reduced cleft width, and enhanced nasal symmetry, whereas others highlight limited or transient benefits, particularly in bilateral cases. ^8^^,^^9^

Previous reviews have mostly focused on nasal outcomes, esthetic aspects, or parental satisfaction, with fewer analyses addressing objective maxillary arch changes. ^10^^,^^11^ Furthermore, the long-term stability of NAM-induced corrections remains unclear, as natural growth patterns and subsequent surgical interventions may alter initial outcomes. In recent years, numerous investigations have evaluated the impact of infant orthopedic interventions-particularly nasoalveolar molding (NAM)-on maxillary arch morphology in patients with cleft lip and palate. Thiruvenkatachari et al. ^12^ reported that, especially in bilateral cleft cases, the NAM appliance often requires frequent adjustments, and its effectiveness may depend heavily on the clinician’s expertise and the degree of parental compliance. Similarly, Magyar et al. in an analysis stratified by cleft severity, demonstrated that therapeutic outcomes tend to be more stable in milder clefts compared to severe ones. ^13^ Moreover, Ocak et al. ^14^, through three-dimensional evaluation of maxillary casts, found that NAM can improve early nasal symmetry and dental arch form, although its influence on the longitudinal dimensions of the maxillary arch appears limited. Consistent with these findings, Chaisooktaksin et al. ^15^ reported that a modified NAM design significantly reduces both anterior and posterior cleft width.

Conversely, other studies have raised concerns regarding specific aspects of transverse maxillary development following NAM therapy. Chang demonstrated that the canine-midline discrepancy between the cleft and non-cleft sides was greater among infants treated with NAM compared with untreated controls, suggesting that NAM may not necessarily improve anterior transverse symmetry and may even increase asymmetry in certain cases. ^2^ Tankittiwat et al. ^16^ further reported that although NAM effectively reduces premaxillary protrusion and rotation in bilateral cleft patients, it has no significant long-term effect on anterior arch width or intercanine distance, indicating limited influence on anterior transverse maxillary dimensions over time.

Earlier investigations have generally supported the short-term benefits of NAM. Saad ^17^ found that NAM reduces cleft severity and facilitates realignment of maxillary segments without producing major adverse effects. Similarly, Chantadilok et al. ^3^ documented significant reductions in anterior cleft width and intercanine distance in infants with unilateral cleft lip and palate following NAM therapy. Comparable results were reported by Singh et al. ^18^, who compared the Grayson and Figurae NAM techniques using digital cast analysis and observed significant reductions in anterior cleft width with both approaches, although the figure technique required notably fewer adjustment visits. Finally, Sabarinath et al. ^19^ demonstrated that NAM not only decreases alveolar cleft width but also significantly increases arch perimeter, supporting the appliance’s role in guiding early maxillary arch development in the anterior region.

Overall, the reviewed literature suggests that NAM-particularly during the early stages of treatment-can positively influence several anatomical and esthetic parameters in infants with cleft lip and palate. However, its long-term impact on maxillary arch morphology remains controversial, highlighting the need for further well-designed studies with standardized protocols and extended follow-up durations.

Given these uncertainties, a systematic review and meta-analysis was undertaken to evaluate both the immediate and long-term effects of NAM on maxillary arch dimensions and morphology in patients with unilateral and bilateral cleft lip and palate. By synthesizing available evidence, this study aims to provide a clearer understanding of the role of NAM in presurgical orthopedic management of cleft patients.

## MATERIALS AND METHODS

The aim of this study was to determine whether nasoalveolar molding (NAM) provides temporary improvement or produces stable changes in maxillary arch growth and orientation. As this study was a systematic review and meta analysis, no direct ethical concerns were involved. The protocol was reviewed and approved by the Ethics Committee of Mashhad School of Dentistry (IR.MUMS.DENTISTRY.REC.1403.039; April 14, 2024).

### Study Design and Guidelines

This systematic review and meta analysis was conducted according to the PRISMA guidelines. The final literature search was completed on July 7, 2025. The research question was structured using the PICO framework: newborn infants with unilateral or bilateral cleft lip and palate (population); nasoalveolar molding initiated during infancy (intervention); infants who did not receive NAM or other infant orthopedic treatments (comparison); and the short and long term effects of NAM on maxillary arch morphology (outcome).

### Eligibility Criteria

Studies were included if they involved human newborns with unilateral or bilateral cleft lip and palate, contained a control group without NAM therapy, and evaluated the effect of NAM or infant orthopedic treatment on maxillary arch morphology. Cohort and analytical interventional studies with available full texts were considered without language or time restrictions. Studies were excluded if outcomes were not relevant or quantifiable, if syndromic clefts or animal models were used, or if they were reviews, case reports, or conference abstracts.

### Search Strategy

A comprehensive search was performed in PubMed, ISI Web of Science, Scopus, EMBASE, and the Cochrane Central Register of Controlled Trials using predefined keywords and their combinations. Titles and abstracts were screened by two independent reviewers, and full texts of potentially eligible studies were assessed. Disagreements were resolved through discussion and consensus.

### Data Extraction

Data extraction was conducted by one reviewer and verified by a second. Extracted variables included authors, publication year, study design, cleft type (UCLP/BCLP), sample size, patient age and sex, study duration, NAM technique, and outcome assessment methods. Any discrepancies were resolved by a third reviewer.

### Risk of Bias Assessment

Randomized clinical trials were evaluated using the Cochrane Risk of Bias 2 (ROB 2) tool across five domains: randomization process, deviations from intended interventions, missing outcome data, outcome measurement, and selective reporting. Non randomized studies were assessed using appropriate methodological criteria for observational studies.

### Statistical Analysis

Meta analysis was performed using RevMan 5.4 software. Standardized mean differences (SMD) with 95% confidence intervals were calculated for outcomes reported in at least two studies. A random effects model was used when heterogeneity was significant (I² > 50% or p < 0.1); otherwise, a fixed effect model was applied. The main outcomes analyzed were anterior alveolar cleft width, posterior arch width, and intercanine width, with statistical significance set at p < 0.05.

## RESULTS

A total of 855 articles were identified through database searches (MEDLINE: 201; ISI Web of Science: 212; EMBASE: 205; SCOPUS: 185; Cochrane Central: 52), along with 26 additional articles identified through hand searching, resulting in 881 records in total. After removing 532 duplicates, the titles and abstracts of 349 articles (retrieved from the databases) were screened. Of these, 295 articles were excluded for not meeting the inclusion and exclusion criteria. The full texts of the remaining 49 studies were then reviewed. Among these:

- 10 studies were excluded because they compared two different NAM techniques,
- 25 studies were excluded because they only assessed pre- and post-treatment outcomes of NAM in a single group without a control group,
- 1 study was excluded due to having a control group consisting of healthy individuals,
- 1 study was excluded because it investigated changes in the nose and face,
- 1 study was excluded as it compared the cleft and non-cleft sides of the maxilla within the same group of infants,
- 2 studies were excluded because they were case reports,
- 3 studies were excluded because, despite having a control group, they did not report pre-treatment values. (Figure 1)

**Figure 1:**
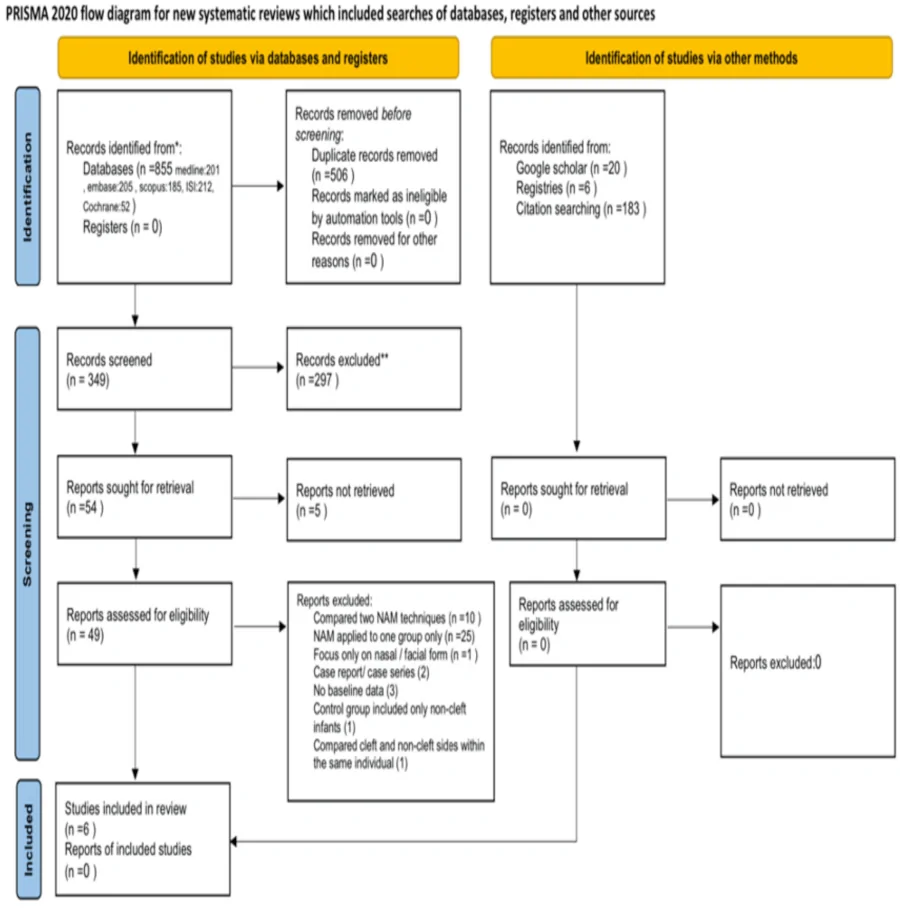
PRISMA flowchart

Finally, 6 studies were included in our systematic review and meta-analysis. Of these, 5 studies involved unilateral cleft lip and palate, and 1 study involved bilateral cleft lip and palate. One study was retrospective. Among the six included studies, only one study assessed the long-term effects of nasoalveolar molding, while the remaining studies investigated only the short-term effects of NAM. 

A total of six studies were included in the systematic review and meta-analysis: five on unilateral cleft lip and palate (UCLP) and one on bilateral cleft lip and palate (BCLP). The studies differed in design, sample size, appliances used, and outcome measures, but all investigated the effects of nasoalveolar molding (NAM) on maxillary arch morphology. (Table 2)

**Table 1:** Databases. Applied search strategy, and numbers of retrieved studies.

Database of published trials, dissertations and conference proceedings	Search strategy used	Hits
MEDLINE searched via PubMed searched on April 29, 2025^th^, via www.ncbi .nlm.nih.gov/sites	((Cleft Palate[MeSH Major Topic]) OR (Cleft lip AND palate) OR cleft lip OR cleft palate OR CL?P OR oral cleft OR orofacial cleft) AND (nasoalveolar molding OR NAM OR Presurgical nasoalveolar orthop?edic OR PNAM) AND (maxil* OR alveol* OR arch) AND (width OR dimension OR measurement OR size)	201
ISI web of science Core Collection was searched via web of knowledge on April 29, 2025^th^, via apps.webofknowledge.com	((Cleft lip AND palate) OR (cleft lip) OR (cleft palate) OR (CL?P) OR (oral cleft) OR (orofacial cleft)) AND ((nasoalveolar molding) OR (NAM) OR (Presurgical nasoalveolar orthop?edic) OR (PNAM)) AND ((maxil* OR alveol* OR arch) AND (width OR dimension OR measurement OR size)) (Topic)	212
EMBASE searched via Ovid on April 29, 2025^th^, via http://ovidsp.dc2.ovid.com	(('cleft lip'/exp OR 'cleft lip' OR (cleft AND ('lip'/exp OR lip))) AND ('palate'/exp OR palate) OR 'cleft lip'/exp OR 'cleft lip' OR (cleft AND ('lip'/exp OR lip)) OR 'cleft palate'/exp OR 'cleft palate' OR (cleft AND ('palate'/exp OR palate)) OR cl?p OR 'oral cleft'/exp OR 'oral cleft' OR (oral AND cleft) OR 'orofacial cleft'/exp OR 'orofacial cleft' OR (orofacial AND cleft)) AND ('nasoalveolar molding'/exp OR 'nasoalveolar molding' OR (nasoalveolar AND ('molding'/exp OR molding)) OR nam OR 'presurgical nasoalveolar' OR (presurgical AND nasoalveolar AND orthop?edic) OR pnam) AND (maxil* OR alveol* OR arch) AND ('width'/exp OR width OR 'dimension'/exp OR dimension OR 'measurement'/exp OR measurement OR 'size'/exp OR size)	205
Scopus searched via Scopus on April 29, 2025^th^, via https://www.scopus.com	TITLE-ABS-KEY (((Cleft lip AND palate) OR (cleft lip) OR (cleft palate) OR (CL?P) OR (oral cleft) OR (orofacial cleft)) AND ((nasoalveolar molding) OR (NAM) OR (Presurgical nasoalveolar orthop?edic) OR (PNAM)) AND ((maxil* OR alveol* OR arch) AND (width OR dimension OR measurement OR size)))	185
Cochrane Central Register of Controlled Trials searched via the Cochrane Library Searched on April 29, 2025^th^, via www.thecochranelibrary.com	((Cleft lip AND palate) OR (cleft lip) OR (cleft palate) OR (CL?P) OR (oral cleft) OR (orofacial cleft)) AND ((nasoalveolar molding) OR (NAM) OR (Presurgical nasoalveolar orthop?edic) OR (PNAM)) AND ((maxil* OR alveol* OR arch) AND (width OR dimension OR measurement OR size))	52
Total		855

- Abd El Ghafour et al. ^20^ (2020, RCT): Thirty-four infants with UCLP were randomized into NAM and control groups. Parameters assessed included anterior cleft width (ACW), posterior cleft width (PCW), intercanine width (ICW), middle arch width (MAW), posterior arch width (PAW), greater and lesser segment lengths, and segment rotation angle. Results indicated significant short-term reduction of ACW, though other parameters showed limited changes.
- Quan Yu et al. ^21^ (2013, RCT): Thirty UCLP infants were evaluated using CAD-NAM. Measurements included ACW, MAW, alveolar arch length (AAL), premaxillary protrusion, and midline deviation. NAM significantly reduced ACW and improved segment alignment, although changes in PAW and AAL were not statistically significant.
- Lautner et al. ^22^ (2019, retrospective): Twenty UCLP infants were treated with conventional NAM and compared with controls. Outcomes included ACW and alveolar cleft volume. NAM significantly reduced ACW and cleft volume before lip repair, demonstrating its role in narrowing cleft gaps, though limited by small sample size and lack of long-term follow-up.
- De Souza et al. ^23^ (2023, RCT): Twenty-six UCLP infants were studied with NAM versus controls. Parameters included arch perimeter, AAL, and the angle of the labial frenulum with the midline. NAM produced modest improvements in arch perimeter and labial frenulum alignment, but group differences were not statistically significant.
- Xin Gong et al. ^4^ (2017, RCT): Thirty-seven BCLP infants were randomized to CAD-NAM or controls. Outcomes assessed included ACW, MAW, and premaxillary protrusion. NAM achieved significant reduction in ACW and midline deviation, but had limited effect on long-term stabilization of maxillary morphology.
- Vikram Shetty et al. ^24^ (2017, RCT): A large cohort of 120 UCLP infants was divided into subgroups based on age at initiation of NAM. Parameters included ACW, ICW, and PAW. Early NAM initiation (<1 month) showed greater reduction in ACW. ICW demonstrated mild reductions, while PAW increased over time, suggesting differential effects of NAM on anterior versus posterior arch dimensions.

In order to provide a clearer overview of the methodologies and findings, the detailed characteristics of the included studies are summarized in Table 3.

In this review, out of six RCTs evaluated, Soza et al. ^23^ exhibited “some concerns” in domain 1 (randomization process), while Gong ^4^ showed “some concerns” in domain 5 (selection of reported results). The remaining domains across both studies were rated as “low risk.” Overall, all studies were judged as either “low risk” or “some concerns,” with no study rated as “high risk.” These findings suggest generally acceptable methodological quality, with minor concerns that may slightly affect internal validity but are not expected to substantially bias the results. (Figure 2)

**Figure 2:**
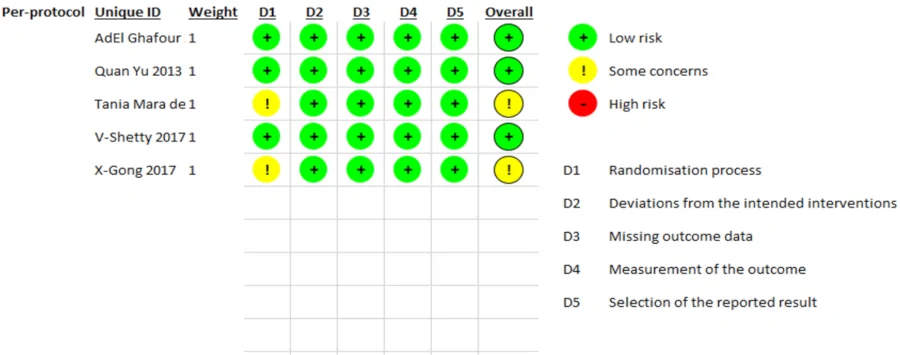
Results of the risk of bias of the RCT studies included in the study assessed using ROB 2

Across the six studies, NAM consistently reduced anterior cleft width in the short term, confirming its effectiveness in narrowing the alveolar gap before lip repair. Effects on ICW, PAW, and other parameters were variable and often non-significant, with heterogeneity linked to cleft type (unilateral or bilateral), timing of initiation, and type of appliance (conventional vs. CAD-NAM). (Figure 3)

**Figure 3:**
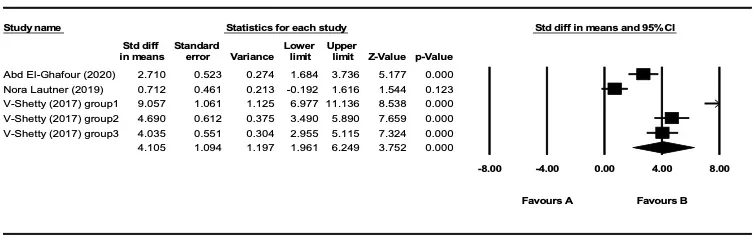
Forest plot for anterior alveolar cleft width.

Three studies (n = 84 NAM, n = 84 control) were included in the meta-analysis of AACW, showing a significant short-term reduction in cleft width in the NAM group (SMD = 4.10; 95% CI = 1.96-6.24; p < 0.001), though heterogeneity was high (I² = 94.1%). (Figures 4, 5)

**Figure 4:**
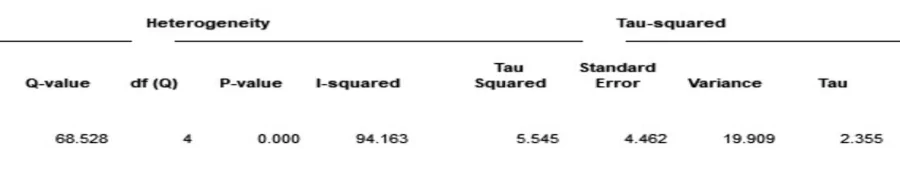
Heterogenisity of studies evaluated for anterior alveolar cleft width.

**Figure 5:**
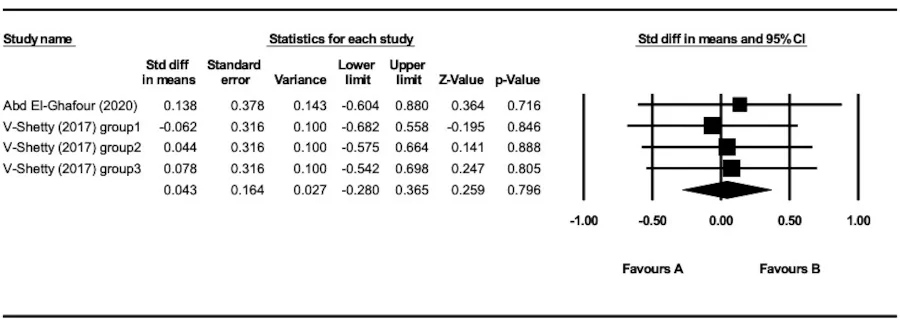
Forest plot for posterior arch width.

For PAW and ICW, two studies (n=74 NAM, n=74 control) provided data. The pooled analysis revealed no statistically significant differences between NAM and control groups (ICW: SMD = 0.043; 95% CI = -0.280 to 0.365; p = 0.796, I² = 0%). Similarly, no consistent effect was found for PAW. (Figures 6-8)

**Figure 6:**
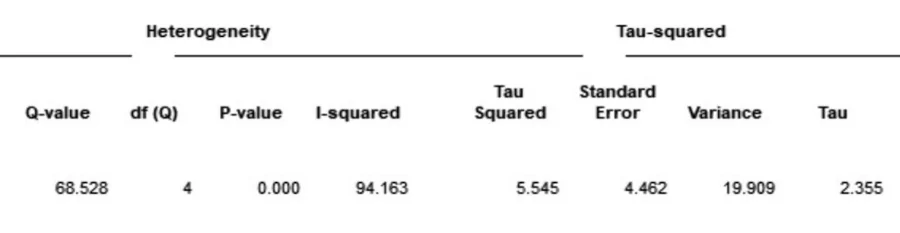
Heterogenisity of studies evaluated for posterior arch width.

**Figure 7:**
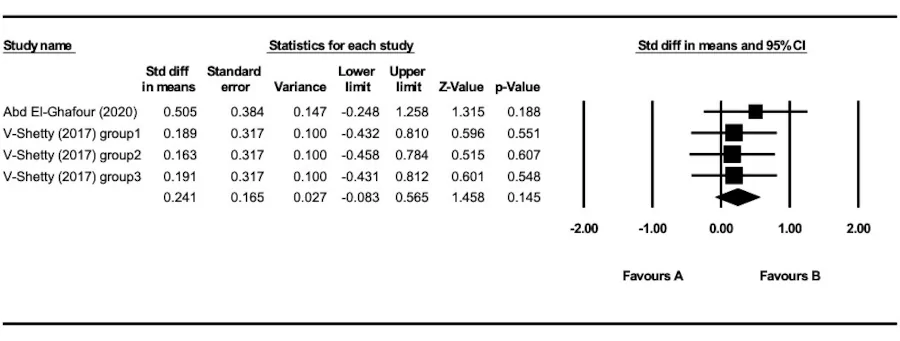
Forest plot for intercanine width.

**Figure 8:**
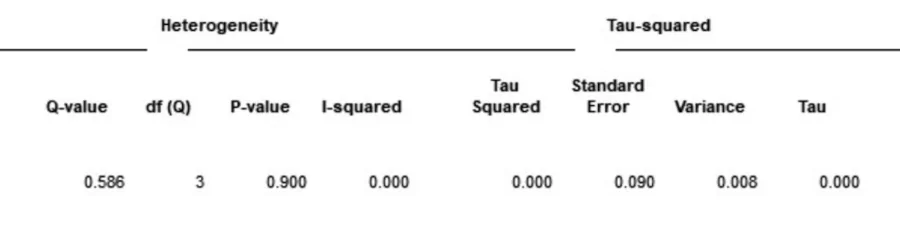
Heterogenisity of studies evaluated for inter canine width.

Long-term outcomes were scarce. Only one study reported follow-up beyond primary surgery, suggesting that while initial improvements in AACW could be maintained for several years, subsequent surgical interventions and individual growth patterns could modify these results.

## DISCUSSION

In this systematic review and meta-analysis, the aim was to evaluate the effectiveness of nasoalveolar molding (NAM) on the dimensions and morphology of the maxillary arch in infants with unilateral or bilateral cleft lip and palate. Six eligible studies were included, each assessing structural changes in maxillary arch anatomy following NAM therapy. A key question was whether NAM should be regarded merely as a presurgical preparation method or whether it has the potential to create meaningful and lasting modifications in early maxillary form and future growth patterns.

Findings showed that the parameter most responsive to NAM was the anterior cleft width (ACW). Five of the six included studies reported a significant reduction in ACW in the NAM group, whereas the control groups showed minimal to no change. Even in studies where the control group consisted of only a single preoperative measurement, the reduction in the NAM group remained substantial. However, long-term stability of these changes is uncertain; as demonstrated in Shetty’s study, the initial reduction in ACW did not remain significant over time. ^10^ These findings suggest that the primary effect of NAM on cleft width is largely short-term and presurgical.

Results regarding inter-canine width (ICW) were inconsistent and heavily influenced by study design. Some studies, such as Abd El Ghafour et al. ^20^, reported no significant differences between groups, while others, including Saad and Shetty, observed a meaningful increase in ICW after NAM therapy. Age at the start of treatment appeared to be a critical factor, with infants treated before one month of age showing different outcomes compared to older infants. Overall, however, the evidence does not strongly support a consistent or predictable effect of NAM on ICW.

Posterior arch width (PAW) was evaluated in three of the included studies, none of which found significant differences between the NAM and control groups. While some studies noted within-group changes after treatment, these did not translate into meaningful differences in comparative analyses. Earlier studies have reported similar patterns, indicating that PAW is one of the parameters least responsive to NAM, and the intervention appears to have limited influence on posterior arch development.

Middle arch width (MAW) also demonstrated no significant differences between groups in the three studies that examined this parameter. These results parallel those of posterior arch dimensions and suggest that NAM exerts its most pronounced effects on the anterior region of the maxillary arch, with minimal impact on middle arch dimensions. In bilateral clefts, the scarcity of available data prevents firm conclusions.

Alveolar arch length (AAL) was assessed in only two studies, both of which indicated no significant effect of NAM. Although increases in arch length were observed in both NAM and control groups, the differences between groups were not statistically meaningful. These findings imply that AAL, like the posterior and middle arch dimensions, is relatively resistant to modification by NAM.

The meta-analysis revealed that NAM’s influence is not uniform across different anatomical parameters. ACW was the only parameter that showed a significant and substantial improvement following NAM therapy, while PAW exhibited no statistically significant changes. The high heterogeneity observed for some parameters reflects variations in study designs, initial cleft severity, age at treatment initiation, and measurement methods. These discrepancies highlight the need for standardized treatment protocols and research methodologies.

Comparisons with recent studies, including those by Magyar et al. ^13^ and Bangun et al. ^25^, reaffirm that NAM primarily affects the anterior region of the maxillary arch and is particularly effective in reducing premaxillary protrusion. However, its impact on posterior dimensions and inter-canine width remains inconsistent. These studies also emphasize practical challenges associated with NAM, such as the importance of early initiation, the degree of initial deformity, parental compliance, treatment cost, and access to specialized centers.

Similarly, the systematic review by Rabal-Solán et al. ^26^ reported no significant differences between intervention and control groups for multiple intra-arch parameters among infants with cleft lip and palate, reinforcing the need for high-quality randomized controlled trials to clarify the true benefits of presurgical orthopedic interventions such as NAM. ^26^

Overall, the findings of this systematic review and meta-analysis indicate that NAM is most effective in reducing anterior cleft width and modifying premaxillary position in the short term. Other maxillary arch dimensions, particularly those in the middle and posterior regions, show minimal or inconsistent response to this intervention. These results suggest that while NAM can play a valuable role in presurgical preparation and facilitate surgical repair, its capacity to induce long-term structural changes in maxillary growth remains uncertain. Future research should incorporate standardized designs, appropriate control groups, long-term follow-up, and larger sample sizes to provide more definitive evidence regarding the effectiveness of NAM.

## LIMITATIONS

This systematic review and meta-analysis should be interpreted in light of several limitations. First, the number of eligible studies was relatively small, and the majority of them included unilateral cleft lip and palate patients, while only a few focused on bilateral cases. This imbalance restricts the ability to generalize findings across different cleft types. Second, heterogeneity existed among the included studies in terms of study design, sample size, treatment protocols, timing of NAM initiation, and measurement techniques, which may have influenced the pooled results. Third, the duration of follow-up was often short, and only one study provided long-term data, limiting the assessment of the stability of NAM effects over time. Moreover, some studies lacked appropriate control groups or presented incomplete data for comparison, which reduced the strength of evidence. Finally, potential biases such as variations in parental compliance, operator experience, and adjunctive surgical interventions could not be fully accounted for and may have affected the outcomes.

## CONCLUSION

This systematic review and meta-analysis demonstrated that nasoalveolar molding provides a significant short-term reduction in anterior arch cleft width in infants with cleft lip and palate, particularly in unilateral cases. However, its effects on other maxillary arch parameters, including intercanine width, posterior arch width, and alveolar arch length, were inconsistent and not statistically significant. Evidence regarding bilateral clefts and long-term outcomes remains scarce and inconclusive, highlighting the need for further high-quality prospective studies. While NAM is effective in facilitating tension-free lip closure and improving pre-surgical alignment, its long-term role in craniofacial growth and stability requires further investigation.

### Clinical Implications

The findings of this review emphasize that nasoalveolar molding can be considered a valuable pre-surgical orthopedic intervention, particularly for reducing the anterior cleft width before primary lip repair in unilateral cleft cases. For bilateral clefts, the available evidence is still limited, and outcomes appear less predictable. Families and cleft care teams should be aware that NAM offers short-term improvements that may facilitate surgery, but its long-term benefits on maxillary growth and symmetry remain uncertain. Expanding multicenter studies with longer follow-up are essential to guide standardized treatment protocols and provide clearer recommendations for practice.

## Source(s) of support:

Self-financed.
